# Inflammation and immunity connect hypertension with adverse COVID-19 outcomes

**DOI:** 10.3389/fgene.2022.933148

**Published:** 2022-09-08

**Authors:** Lei Cai, Chuan He, Yonglin Liu, Yanlan Sun, Lin He, Ancha Baranova

**Affiliations:** ^1^ Bio-X Institutes, Key Laboratory for the Genetics of Developmental and Neuropsychiatric Disorders (Ministry of Education), Collaborative Innovation Center for Genetics and Development, Shanghai Mental Health Center, Shanghai Jiaotong University, Shanghai, China; ^2^ Sanya Women and Children’s Hospital managed by Shanghai Children’s Medical Center, Sanya, China; ^3^ Shanghai Center for Women and Children’s Health, Shanghai, China; ^4^ School of Systems Biology, George Mason University, Fairfax, VA, United States

**Keywords:** COVID-19, hypertension, DBP, SBP, meta-analysis, multiple sclerosis

## Abstract

**Objectives:** To explore the connection of hypertension and severe COVID-19 outcomes.

**Methods:** A total of 68 observational studies recording mortality and/or general severity of COVID-19 were pooled for meta-analyses of the relationship of severe COVID-19 outcomes with hypertension as well as systolic and diastolic blood pressure. Genome-wide cross-trait meta-analysis (GWCTM) was performed to explore the genes linking between hypertension and COVID-19 severity.

**Results:** The results of meta-analysis with the random effect model indicated that pooled risk ratios of hypertension on mortality and severity of COVID-19 were 1.80 [95% confidence interval (CI) 1.54–2.1] and 1.78 (95% confidence interval 1.56–2.04), respectively, although the apparent heterogeneity of the included studies was detected. In subgroup analysis, cohorts of severe and mild patients of COVID-19 assessed in Europe had a significant pooled weighted mean difference of 6.61 mmHg (95% CI 3.66–9.55) with no heterogeneity found (*p* = 0.26). The genes in the shared signature of hypertension and the COVID-19 severity were mostly expressed in lungs. Analysis of molecular networks commonly affected both by hypertension and by severe COVID-19 highlighted CCR1/CCR5 and IL10RB signaling, as well as Th1 and Th2 activation pathways, and also a potential for a shared regulation with multiple sclerosis.

**Conclusion:** Hypertension is significantly associated with the severe course of COVID-19. Genetic variants within inflammation- and immunity-related genes may affect their expression in lungs and confer liability to both elevated blood pressure and to severe COVID-19.

## Introduction

The coronavirus disease 2019 (COVID-19) pandemic, which is caused by severe acute respiratory syndrome coronavirus 2 (SARS-CoV-2), has created a public health crisis worldwide. This disease is associated with a wide spectrum of clinical manifestations ranging from mild to life-threatening, with a possibility of adverse outcomes. Across 2 years, the prevention and therapy went through a few rounds of optimization, which decreased the global fatality rate for COVID-19 to 1.22% (COVID-19. who.int/). Further improvements require comprehensive understanding of COVID-19 pathogenesis through elucidating both the viral and the host determinants of the severe course of COVID-19.

Hypertension is a common disease defined as the systolic blood pressure (BP) readings on two different days being over 140 mmHg, and/or the diastolic BP readings ≥90 mmHg (Organization, 2019). While hypertension is repeatedly reported as one of the predictors of adverse SARS-CoV-2 infection outcomes in various cohorts (Manohar, 2021; Stanetic et al., 2021; Kumaran et al., 2022), the connection of this multifactorial condition and severe COVID-19 remains not very well characterized, especially when compared to that of obesity or diabetes (Madjid et al., 2020; Schiffrin, 2020). Relative lack of systemic efforts in clinical dissection of interactions between hypertension and COVID-19 is surprising, especially in light of an active involvement of ACE2, a receptor for SARS-CoV-2, in the hypertension control (Verano-Braga et al., 2020). On the other hand, an importance of hypertension as a factor contributing to COVID-19 outcomes is underlined by notions that adequate control of BP is one of the prerequisites for alleviating COVID-19-associated organ damage (Zheng et al., 2020; Hessami et al., 2021; Wu et al., 2021).

The genome-wide association study (GWAS) is a powerful tool to identify genetic variants contributing to complex phenotypic traits, which are influenced by many factors at once. Under the assumption that many gene variants may be associated with multiple traits, cross-phenotype (CP) associations analyses were made possible recently by introducing the CPASSOC package that uses summary-level data from GWAS to analyze multiple phenotypes for each SNP by accounting for their correlations among traits and among cohorts (Park et al., 2016; Li and Zhu, 2017). We mined multiple existing GWASs that have reported various outcomes related to COVID-19, including hospitalization, severe respiratory problems, and even death in an attempt to extract a list of genes possibly contributing to hypertension and the severity of COVID-19. We analyzed the functions of these genes and extracted additional insights into common pathophysiology of these two diseases.

## Methods and materials

### Literature search strategy

An extensive search was performed within the databases of PubMed, COVID-19 Portfolio, Embase, Scopus, and China National Knowledge Infrastructure (CNKI) by using the keywords of (COVID-19 OR SARS-CoV-2 OR coronavirus) AND (severity OR clinical outcome) AND (hypertension OR blood pressure). All relevant sources from 30 December 2019 to 20 June 2021 were retrieved without language restrictions. Two authors (CH and YS) independently reviewed the collected literature works. Furthermore, the reference lists of all relevant studies were also manually checked for additional entries.

### Study selection and evaluation

Eligible studies were considered when they met the following criteria: 1. there was properly established COVID-19 diagnosis; 2. complete data for survivor/non-survivor or severe COVID-19 infection/mild; 3. complete data for hypertension prevalence or BP measurement; and 4. complete information for subject characteristics. Here, COVID-19 diagnosis was based on SAR-CoV-2 reverse transcription-polymerase chain reaction (RT-PCR) testing of nasopharyngeal (NP) swab, throat swab, or other types of respiratory sampling, and the severe COVID-19 infection was detected in patients requiring oxygen support, or admitted to intensive care, or reported as dead. In case of any degree of non-clarity in the data, the corresponding authors were contacted for full information. The exclusion criteria were as follows: 1. duplicated studies; 2. meta-analyses, reviews, case reports, and nonhuman studies; 3. containing COVID-19 patients with pneumonia or other lung diseases; 4. containing pediatric patients; and 5. the quality assessment scores of studies were below 7. The quality score of each study was less than 7 based on the principles of AHRQ (Rostom, 2004) and QUADAS (JB, 2003), which was assessed based on five items (Supplementary Table S1). The third party (LC and YL) took part in discussion to solve the disagreement of the evaluation result.

### Data extraction

The following information was extracted from each study: first author’s name, publication year, period of patients’ admission, study type, country, sample size, age, gender, hypertension prevalence, systolic blood pressure, diastolic blood pressure, cases of non-survivors or severe COVID-19 infection, and controls of non-survivor of mild COVID-19 infection were recorded into a standardized information sheet.

### Genome-wide cross-trait meta-analysis

The cross-phenotype association (CPASSOC) approach (Zhu et al., 2015) was employed to identify genetic variants shared between COVID-19 and hypertension. CPASSOC allows for the presence of heterogeneous effects across traits and provides S_Het_ statistics and *p*-values weighted by a sample size. In two-step CPASSOC analysis, the correlation matrix was built with SNPs whose summary statistics Z-scores were greater than 1.96 or less than −1.96 and which had a linkage disequilibrium (LD) pattern from 1,000 Genomes Project phase 3, and then S_Hom_ and S_Het_ tests were performed. S_Hom_ is more powerful when heterogeneity is not present, while S_Het_ allows for trait heterogeneity. Significant levels of less than 5 × 10^–8^ were used as cut-off values. Here, λmeta statistics were calculated to test the possibility of sample overlap through measuring concordance of effect sizes (Chen et al., 2016). Under the null hypothesis, λmeta = 1 means that the pair of cohorts are completely independent, while λmeta <1 indicates samples overlap between cohorts.

### Identification of shared genes and enrichment analysis

The FUMA (v1.3.5 d) (https://fuma.ctglab.nl/), a platform for annotation, visualization, and interpretation of GWAS results, was utilized for functional mapping of the genes found by GWCTM (Watanabe et al., 2017), with the summary statistics obtained from GWCTM treated as input. In the beginning, positional gene mapping of SNP2GENE function within FUMA was carried out. Then, the tissue specificity of these genes was explored with GENE2FUNC within FUMA and GTEx v8 54 tissue-types data. Finally, the eQTL gene mapping was carried out within the aforementioned identified specific tissue of the GTEx v8 dataset.

IPA (Ingenuity Pathway Analysis) software (Ingenuity Systems; Qiagen China Co., Ltd.) was employed to perform core enrichment analysis in human lung tissue with genes identified for COVID-19 severity and its adverse outcomes, respectively. The Gene Ontology (GO) enrichment analysis was performed as previously described (Cai et al., 2018).

### Statistical analysis

For studies reporting the interquartile range, the standard deviation values were obtained as described in the Cochrane Handbook for Systematic Reviews (Cumpston et al., 2019). The weighted mean differences (WMDs) were calculated with 95% confidence intervals (CIs) (Cai et al., 2015). The heterogeneity was evaluated using the Q-test and I^2^ statistic (Cai et al., 2013). I^2^ > 50% or *p* < 0.1 were considered significantly heterogeneous. A fixed-effects model was used when the result showed no significant heterogeneity; otherwise, a random-effects model was applied. The publication bias was evaluated by Egger’s regression test (Lei et al., 2005). All the analyses were conducted in accordance with the Cochrane Handbook for Systematic Reviews (Version 5.0) or R software, with two-sided *p* < 0.05 set for statistical significance.

## Result

### Data collection

The process of literature selection is shown in Supplementary Figure S1. Through electronic-database searching and manual examination of the reference lists, we collected a total of 4,284 records. After three rounds of filtering, a total of 68 studies were retained for further statistics analysis, among which there were 21 studies containing 32,015 COVID-19 patients with the outcome of survivor/non-survivor, and 47 studies including 230,941 COVID-19 patients with the outcome of clinical severity or mildness. The main characteristics of studies included in the current meta-analysis are summarized in Table 1.

**TABLE 1:** Characteristics of studies included in the current meta-analysis.

Study	Period	Outcome	Country	Number of subjects		Age (year)		Gender (male %)		Number of hypertension	
				Case	Control	Case	Control	Case	Control	Case	Control
Souza, et al., 2021	March–May 2020	Dead/survivor	India	156	533	55 ± 29.9	38 ± 29.7	60.26	45.78	33	33
Surendra, et al., 2021	March–July 2020	Dead/survivor	Indonesia	497	3,768	58.7 ± 11.9	43.3 ± 18	61	51	184	594
Pareek, et al., 2021	March–May 2020	Dead/survivor	United States of America	82	504	78.8 ± 15.8	65.7 ± 17.8	63	50.8	60	293
Muhammad, et al., 2021	March–May 2020	Dead/survivor	United States of America	45	155	67.1 ± 13.7	58 ± 14.9	64.4	59.4	35	95
Marcolino, et al., 2021	March–September 2020	Dead/survivor	Brazil	439	1,551	70 ± 16.4	56 ± 17.8	54.4	51.8	310	746
Kim, et al., 2021	February–July 2020	Dead/survivor	Korea	179	2075	78.3 ± 10.5	54.2 ± 21.5	53.1	34.3	112	534
Halem, et al., 2020	March–April 2020	Dead/survivor	Belgium	81	238	81 ± 7.5	69.6 ± 14.9	61.7	57.98	61	101
Thompson, et al., 2020	March–May 2020	Dead/survivor	United Kingdom	169	301	78.3 ± 12.1	63.4 ± 17.6	54.4	54.1	101	117
Rodriguez-Nava, et al., 2021	March–May 2020	Dead/survivor	United States of America	101	212	74.4 ± 12.8	65.8 ± 14.2	64.4	55.2	79	143
Huang, et al., 2020	January–April 2020	Dead/survivor	China	140	536	66.3 ± 13.5	50.3 ± 20.8	69.3	40.5	84	142
Bonnet, et al., 2021	February–April 2020	Dead/survivor	France	360	2,503	80.4 ± 12	64.6 ± 16.7	61.7	57.3	261	1,186
Diebold, et al., 2021	February–May 2020	Dead/survivor	Switzerland	88	855	75.4 ± 11.3	63.4 ± 17.1	72	72	59	388
Basu, et al., 2021	March–May 2020	Dead/survivor	United Kingdom	361	546	77.8 ± 12.4	66.3 ± 17.6	62	50	287	343
Novelli, et al., 2021	February–March 2020	Dead/survivor	Italy	171	337	75.2 ± 9.2	61.7 ± 16.5	79.5	68.8	120	151
Wang, et al., 2020	January–February 2020	Dead/survivor	China	116	177	72.7 ± 12.1	49.5 ± 22.2	56	41.2	66	26
Zhou, et al., 2020	December 2019–January 2020	Dead/survivor	China	54	137	69.4 ± 9.9	51.6 ± 9.7	70	69	26	32
Huang, et al., 2020	January–March 2020	Dead/survivor	China	16	283	69.2 ± 9.7	52.5 ± 16.6	68.8	52.7	11	63
Iaccarino, et al., 2020	March–April 2020	Dead/survivor	Italy	188	1,403	79.6 ± 0.8	64.7 ± 0.4	66.5	63.6	137	737
Russo, et al., 2020	February–April 2020	Dead/survivor	Italy	35	157	77 ± 8.3	65.5 ± 15.6	57.1	60.5	27	84
Wang, et al., 2020	January–February 2020	Dead/survivor	China	19	277	65.6 ± 12.6	46.0 ± 14.4	57.9	46.6	9	33
Rodilla, et al., 2021	March–June 2020	Dead/survivor	Spain	2,606	9,564	79.7 ± 10.5	64.1 ± 15.7	62.2	55.2	1842	4,352
Thibeault, et al., 2021	March–June 2020	Severe/mild	German	71	90	57.6 ± 22.6	62.7 ± 13.6	70.4	61.1	45	38
Shen, et al., 2021	January–February 2020	Severe/mild	China	32	291	49.78 ± 17	62.22 ± 14.68	56.25	52.58	11	51
Pouw, et al., 2021	March–May 2020	Severe/mild	Netherland	476	476	67.6 ± 16.4	29.3 ± 11.9	68.3	58.8	195	179
Otoshi, et al., 2021	April–November 2020	Severe/mild	Japan	46	254	62.6 ± 17.3	72.2 ± 10.3	30.3	42.9	25	96
Mollinedo-Gajate, et al., 2021	March–April 2020	Severe/mild	Spain	62	131	62.6 ± 15	67.5 ± 16.3	64.5	52.7	30	56
	July–August 2020	Severe/mild	Spain	24	59	69.9 ± 28.1	63.6 ± 27	70.8	52.5	14	26
Zhang, et al., 2021	January–February 2020	Severe/mild	China	51	121	42.3 ± 17.2	60.6 ± 13.7	58.8	51.2	16	16
Scho¨nfeld, et al., 2021	March–October 2020	Severe/mild	Argentina	13,389	193,690	40 ± 18.5	70.65 ± 15.6	58.1	50.6	6,981	32,852
Vlachos, et al., 2021	February–March 2020	Severe/mild	United Kingdom	76	353	68 ± 20.8	55.9 ± 11.3	66	52	38	187
Sim, et al., 2020	February–May 2020	Severe/mild	Malaysia	471	5,418	31.3 ± 10.4	57.6 ± 12.6	71.5	71.7	229	702
Matangila, et al., 2020	March–July 2020	Severe/mild	Congo	19	92	48.9 ± 19.6	51.4 ± 22.4	53	45	10	24
Vial, et al., 2020	March–April 2020	Severe/mild	Chile	18	70	47.4 ± 15.1	66.3 ± 10.5	83.3	40	10	15
Mutair, et al., 2020	April–May 2020	Severe/mild	Saudi Arabia	160	241	37.32 ± 13.6	39.43 ± 13.1	81.3	79.3	33	26
He, et al., 2020	January–March 2020	Severe/mild	China	501	530	57.5 ± 15.6	65.3 ± 11.9	59.3	52.2	237	146
Xiong, et al., 2020	February–March 2020	Severe/mild	China	55	61	52.1 ± 20.5	64.4 ± 17.5	69.1	68.9	26	19
Jourdes, et al., 2020	March–April 2020	Severe/mild	France	50	213	64.4 ± 17.1	65.2 ± 13	66	57.3	19	85
Popov, et al., 2020	March–June 2020	Severe/mild	Bulgaria	43	95	48.3 ± 15.7	63 ± 12.8	76.8	56.9	32	37
Nachega, et al., 2020	March–July 2020	Severe/mild	Congo	191	575	42.7 ± 16.4	55.9 ± 14.4	71.1	63.8	87	107
Wei, et al., 2020	January–March 2020	Severe/mild	China	14	262	48.6 ± 13.4	66 ± 10.5	71.4	55.3	8	39
Guan, et al., 2020	January 2020	Severe/mild	China	173	926	45.4 ± 17.1	52.4 ± 16.7	57.8	58.2	41	124
Yue, et al., 2020	January–February 2020	Severe/mild	China	44	42	41.7 ± 20.2	42.5 ± 17.2	52.3	35.7	5	1
Charlotte, et al., 2020	March–April 2020	Severe/mild	Switzerland	49	147	72.6 ± 16.5	63.9 ± 11.5	61	60	27	91
Wang, et al., 2020	January–February 2020	Severe/mild	China	45	230	46.1 ± 21.6	62.1 ± 13	57.8	44.3	18	36
Wang, et al., 2021	January–February 2020	Severe/mild	China	25	122	40.9 ± 13.5	52.5 ± 16.5	76	58.2	8	11
Petrilli, et al., 2020	March–April 2020	Severe/mild	United States of America	990	1739	59.6 ± 17.1	68 ± 14.8	66.3	58.4	680	1,013
Taha, et al., 2021	March–April 2021	Severe/mild	Egypt	50	130	51.1 ± 59.2	57.5 ± 52.7	44	31.5	29	36
Huang, et al., 2020	January 2020	Severe/mild	China	13	28	49.2 ± 12.9	50.5 ± 16.6	85	68	2	4
Krishna, et al., 2021	March–August 2020	Severe/mild	United States of America	70	109	58.6 ± 17.3	62.8 ± 20.4	46	60	37	47
Padmaprakash, et al., 2021	April–August 2020	Severe/mild	India	175	1,361	57	35	80.5	89.3	59	71
Pandita, et al., 2021	February–May 2020	Severe/mild	United States of America	91	168	60.4 ± 17.2	65.4 ± 13.8	55	52.4	63	101
Alhumaid, et al., 2021	March–July 2020	Severe/mild	Saudi Arabia	205	809	45.3 ± 19.3	52.9 ± 17.3	56.5	57.6	111	165
Nasir, et al., 2021	February–June 2020	Severe/mild	Pakistan	137	156	42.3 ± 16.2	59.4 ± 14.1	76.6	55.1	72	30
Neto, et al., 2021	March–May 2020	Severe/mild	Brazil	300	206	58.4 ± 15.5	61.2 ± 14.7	58.7	55.3	168	63
Ser, et al., 2021	April 2020	Severe/mild	Spain	20	42	80.5 ± 3.2	82.9 ± 4.2	60	28.6	12	19
Khan, et al., 2020	March 2020	Severe/mild	Saudi Arabia	77	571	33 ± 18	37 ± 27	67.5	50.8	16	59
Sami, et al., 2020	February–December 2020	Severe/mild	Iran	90	400	55.52 ± 14.45	61.32 ± 16.99	73	58	36	135
Xiong, et al., 2020	January–March 2020	Severe/mild	China	65	407	41.6 ± 14.9	51 ± 21.2	58.5	52.1	22	49
Mughal, et al., 2020	March–April 2020	Severe/mild	United States of America	30	99	56.8 ± 24.8	64.4 ± 10.9	83.3	56.6	14	42
Claudia, et al., 2020	February–April 2020	Severe/mild	Switzerland	35	64	65.3 ± 15.2	66.9 ± 13.9	80	53	19	37
Li, et al., 2020	January–February 2020	Severe/mild	China	269	279	55 ± 16.4	63.6 ± 13.4	56.9	45.2	104	62
Ren, et al., 2020	January–February 2020	Severe/mild	China	62	89	53.9 ± 16.2	67.6 ± 11.6	64.5	42.7	35	25
Cheng, et al., 2020	January–March 2020	Severe/mild	China	52	200	45.4 ± 15.5	62.9 ± 16	59.6	55	20	28
Huang, et al., 2020	January–February 2020	Severe/mild	China	23	179	43.3 ± 14.9	47.6 ± 19	73.9	55.3	2	29
Chen, et al., 2020	January–March 2020	Severe/mild	China	43	102	45.3 ± 13.6	52.8 ± 15.5	53.5	54.9	9	13
Wang, et al., 2020	January 2020	Severe/mild	China	36	102	50.8 ± 5	67.1 ± 16.2	54.3	52	21	22
Zhang, et al., 2020	January–February 2020	Severe/mild	China	58	82	51.6 ± 10.7	62.7 ± 13.5	56.9	46.3	22	20
Gao, et al., 2020	January–February 2020	Severe/mild	China	15	28	52.96 ± 14	45.2 ± 7.68	60	60.7	6	7
Lendorf, et al., 2020	March–May 2020	Severe/mild	Denmark	20	91	69.8 ± 16.6	62.9 ± 15.2	85	55	9	29

GWAS summary datasets for hypertension and COVID-19 are listed in Table 2. Briefly, we utilized one GWAS dataset on hypertension with the largest sample size as found in the MR-base database (Elsworth, 2020) and three GWAS datasets on COVID-19 of varying severity, including recorded cases of death, confirmed very severe respiratory COVID-19, and hospitalized COVID-19.

**TABLE 2:** GWAS summary dataset information.

Data	Source	Trait	Population	Case	Control	N
Hypertension	https://gwas.mrcieu.ac.uk/datasets/ukb-b-12493/	Essential (primary) hypertension, SBP >140 or DBP >90 vs. population	European	54,358	408,652	463,010
COVID-19 death	https://grasp.nhlbi.nih.gov/	Positive and dead COVID-19 vs. population	European	1,001	458,249	459,250
COVID-19 severe respiration	https://www.covid19hg.org/	Very severe respiratory confirmed COVID-19 vs. population	European	4,792	1,054,664	1,059,456
COVID-19 hospitalization	https://www.covid19hg.org/	Hospitalized COVID-19 vs. population	European	9,986	1,877,672	1,887,658

### Association of severity of COVID-19 and hypertension

In mortality meta-analysis of 16,548 survivors and 3,297 non-survivors, the random-effects model was optimal. Using this model, hypertension had an estimated pooled RR of 1.80 (95% CI 1.54–2.1) for COVID-19-related death, with heterogeneity being detected (I^2^ = 91%, *p* < 0.001). With univariable meta-regression model and 1,000 permutations in the permutation test, both age and gender displayed significant influence on the pooled RR, *p* = 0.001 and 0.015, respectively. Taking separately, age and gender regression models explained 66.76 and 26.29% of heterogeneity, respectively. Interestingly, in each sequentially increasing age bracket, the RR of hypertension was decreasing by 0.03 (95% CI −0.043 to −0.019).

In meta-analysis of 19,011 severe and 211,930 mild cases of COVID-19, the random-effects model detected hypertension-related pooled RR of 1.78 (95% CI 1.56–2.04) for severe course of COVID-19, also with evidence of heterogeneity (I^2^ = 97.2%, *p* < 0.001). With univariable meta-regression model and 1,000 permutations in the permutation test, age, but not gender had significant influence on the pooled RR, with *p* = 0.009 and 0.25, respectively. Taking separately, age and gender regression models explained 18.00 and 2.92% of heterogeneity, respectively. Similar to that observed in mortality analysis, in each sequentially increasing age bracket, the RR of hypertension was decreasing by 0.02 (95% CI −0.036 to −0.007).

For both types of analyses, mortality, and severity, the likelihood ratio tests pointed at better fits provided by the univariable meta-regression models than multivariable meta-regression models. For the mortality study, the sensitivity analysis confirmed that omitting any one study had no effect on the pooled RR (Supplementary Figure S2A), with no risk of publication bias (*p* > 0.05). For the severity study, Schȍnfeld’s study performed differently than others, and its omission affected RR (Supplementary Figure S2B), with risk of publication bias detected (*p* = 0.03).

### Association of severity of COVID-19 with blood pressure

**FIGURE 1:**
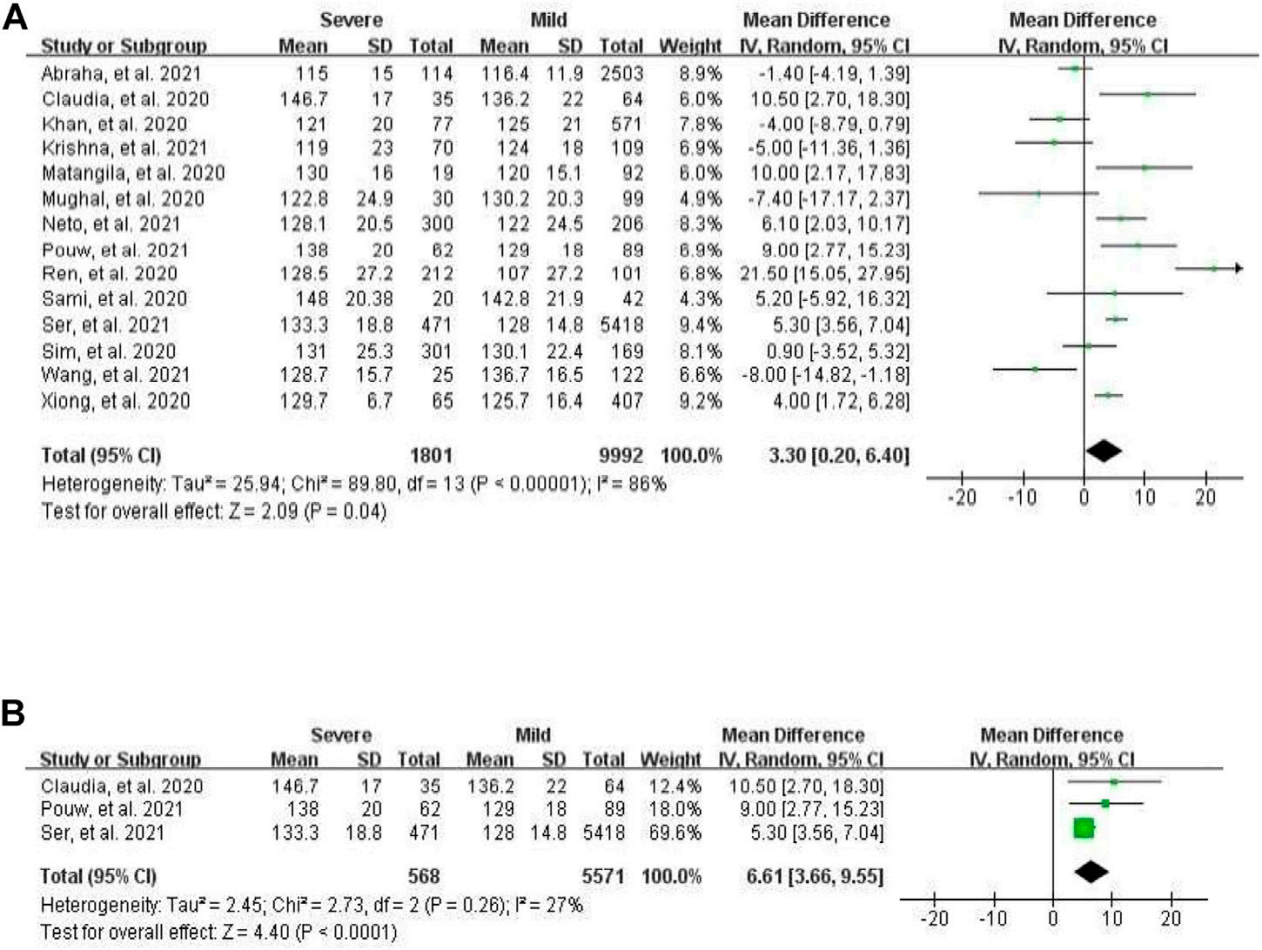
Forest plot for the meta-analysis of mean difference of SBP between severe and mild cases. **(A)** In all populations; **(B)** in the European population.

For systolic blood pressure (SBP), a total of 14 studies, which profiled cohorts with either severe or mild course of COVID-19, were analyzed. The pooled weighted mean difference between these two cohorts was significant at 3.30 mmHg (95% CI 0.20–6.40), with the random-effects model pointing at evident heterogeneity (I^2^ = 86%, *p* < 0.0001, Figure 1A). In further subgroup analysis of three European studies, there was significant pooled weighted mean difference of 6.61 mmHg between severe and mild patients (95% CI 3.66–9.55), with no apparent heterogeneity (I^2^ = 27%, *p* = 0.26, Figure 1B). When a total of nine mortality studies were analyzed, the pooled weighted mean difference between survivors and non-survivors was at -1.54 mmHg (95% CI -4.33 to 1.24), with moderate heterogeneity (I^2^ = 58%, *p* = 0.01).

For diastolic blood pressure (DBP), a total of 13 studies, which profiled cohorts of patients with either severe or milder form of COVID-19, were analyzed. The pooled mean difference between mild and severe groups was −0.44 mmHg and non-significant (95% CI −1.52 to 0.64), with moderate heterogeneity (I^2^ = 45%, *p* = 0.04). In four studies presenting DBP values of deceased sufferers and survivors, the pooled weighted mean difference was 2.95 mmHg and non-significant (95 percent CI −3.28–9.18), with evidence of heterogeneity (I^2^ = 91%, *p* < 0.0001).

### The shared genes between severe course of COVID-19 and hypertension

In the analyzed GWAS dataset, λmeta values were 1.06 ± 0.01, 1.04 ± 0.01, and 1.10 ± 0.01 between hypertension and three adverse COVID-19 outcomes, i.e., death, very severe respiratory problems, and hospitalization. CPASSOC-driven genome-wide cross-trait meta-analysis was performed to search for a common variant contributing both to hypertension and to adverse COVID-19 outcomes, including death, very severe respiratory problems, and hospitalization.

**FIGURE 2:**
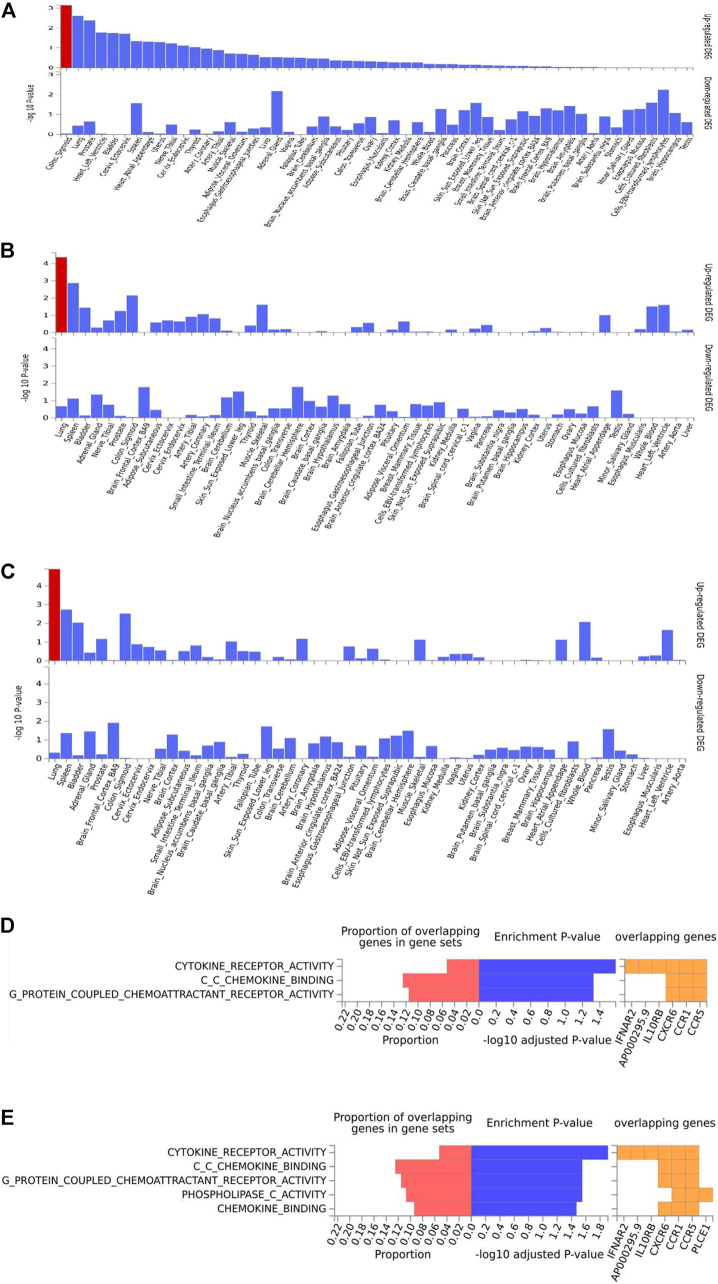
Enrichment analysis of shared genes between hypertension and adverse outcomes of COVID-19, i.e., death, very severe respiration, and hospitalization. **(A–C)** Tissue enrichment of shared genes between hypertension and COVID-19 death, very severe respiration, and hospitalization, respectively. **(D)** and (E) GO function enrichment of shared genes between hypertension and COVID-19 very severe respiration and hospitalization, respectively.

The summary statistics from the aforementioned GWCTM results were further explored by GENE2FUNC implemented in the platform of FUMA. A total of 149 genes were identified as shared between hypertension and COVID-19 death by positional gene mapping of SNP2GENE function (Supplementary Figure S3A); a total of 222 genes were identified as shared between hypertension and very severe, respiratory confirmed COVID-19 by positional gene mapping of SNP2GENE function (Supplementary Figure S3B); and a total of 187 genes were identified as shared between hypertension and the outcomes of the requirement of oxygen support and hospitalization (Supplementary Figure S3C). In both shared gene lists, the top positions were occupied by *CLCN6*, *MTHFR*, *C10orf107*, *FES*, and *FURIN*. Gene expression analysis of shared gene sets highlighted the sigmoid colon as the most relevant to the association of COVID-19 death (Figure 2A), suggesting that the disruption of gut homeostasis in the course of COVID-19 (Varshney et al., 2021) may contribute to COVID-19 mortality disproportionally.

On the other hand, a similarly executed analysis pointed at the lung tissue as the most relevant association of hypertension with other severe COVID-19 outcomes (Figures 2B,C). After integrating GWAS results with lung eQTL data from GTEx (version 8), a total of 67 protein-coding genes were pinpointed as shared between hypertension and the severe respiratory involvement in COVID-19 (Supplementary Table S2) and a total of 57 genes were shared between hypertension and the hospitalization due to COVID-19 (Supplementary Table S3). A majority of these genes were involved in immune function, with cytokine receptor pathways, chemokine binding, and the signaling by G-protein-coupled chemoattractant receptors being particularly highlighted (Figures 2C,D).

The analysis of top canonical pathways identified three common sets of shared genes, namely, pathogenesis of multiple sclerosis, IL-10 signaling, and Th1 and Th2 activation (Table 3). Furthermore, these three sets of genes were enriched within the sets describing some other relevant pathophysiological conditions, including “infectious diseases,” “organismal injury and abnormalities,” “cellular function of altering cell morphology,” and “connective tissue development.”

**TABLE 3:** Top Canonical pathways identified with IPA.

Name	*p*-value	Overlap	Gene
Genes for hypertension with COVID-19 hospitalization			
Pathogenesis of multiple sclerosis	4.38E-04	22.2% (2/9)	CCR1 and CCR5
IL-10 signaling	1.65E-03	4.5% (3/66)	CCR1, CCR5, and IL10RB
Th1 and Th2 activation pathway	1.95E-03	2.7% (4/150)	CCR1, CCR5, CXCR6, and IL10RB
G-protein-coupled receptor signaling	2.37E-03	1.3% (7/523)	CCR1, CCR5, CHP1, CXCR6, MAP3K11, NPR3, and PLCE1
Cardiac hypertrophy signaling (enhanced)	5.29E-03	1.3% (6/455)	ACE, CHP1, IL10RB, MAP3K11, PLCE1, and WNT2B
Genes for hypertension with COVID-19 very severe respiration			
Pathogenesis of multiple sclerosis	5.61E-04	22.2% (2/9)	CCR1 and CCR5
IL-10 signaling	2.36E-03	4.5% (3/66)	CCR1, CCR5, and IL10RB
Th1 and Th2 activation pathway	3.08E-03	2.7% (4/150)	CCR1, CCR5, CXCR6, and IL10RB
Th2 pathway	1.24E-02	2.5% (3/120)	CCR1, CCR5, and CXCR6
Oxytocin signaling pathway	1.46E-02	1.7% (4/235)	CHP1, MFN2, NOS3, and NPR3

## Discussion

Blood pressure is commonly recorded as two numbers: one for systolic BP representing the pressure in blood vessels when the heart contracts or beats, and the other for diastolic BP representing the pressure in the vessels when the heart rests between beats. Hypertension, i.e., elevated blood pressure, is a chronic and serious medical condition that significantly increases the risks of disease of heart, brain, kidney, and other organs. Since hypertension co-occurring with COVID-19 is reported as a risk factor for severe clinical outcomes (Hessami et al., 2021), understanding the association between these two conditions and their mechanistic underpinnings remains a priority. Due to confounding factors, observational studies with a limited sample size may not produce robust results. Thus, we explored the relationship between blood pressure and two types of COVID-19 outcomes, i.e., mortality and severe course of the disease, in a meta-analysis of the existing literature. Despite apparent heterogeneity of the studies included, the random-effects model of the meta-analysis suggested that both for mortality and for severity of COVID-19, their associations with hypertension were significant and substantial, with RR of 1.80 and 1.78, respectively. In mortality meta-analysis, age and gender explained the major share of heterogeneity, while in the meta-analysis of severe COVID-19, the heterogeneity was majorly due to other confounding factors rather than age and gender, thus suggesting that some other parameters should be recorded for studies aiming at exploring relationships of elevated BP with outcomes of COVID-19. In the subgroup meta-analysis of the European populations using the random-effects model, relationships between the severe course of COVID-19 and SBP were significant, with a pooled weighted mean difference of 6.61 mmHg between COVID-19 cohorts with the severe and mild course of illness and no heterogeneity. These findings were in contrast to the analysis of the entire dataset, which detected a significant pooled weighted mean difference of 3.30 mmHg between COVID-19 cohorts with the severe and mild course of illness, with apparent heterogeneity. These observations suggest that elevated BP is significantly associated with the COVID-19 severity, in presence of complex, yet-to-be identified confounding factors.

In further analysis of the gene sets shared between hypertension and severe outcomes of COVID-19, the mainly affected tissue compartment was in the lungs. Thus, the relevant genes were identified in the lungs by integrating GWCTM results with lung eQTL. In the pathway enrichment analysis, the only pathway directly related to the regulation of BP was one involved in cardiac hypertrophy, with its fifth place among top shared pathways connecting hypertension with COVID-19 severity. Instead, predominant involvement of the signaling related to immune function was highlighted, with an emphasis on CCR1 and CCR5, the members of the beta chemokine receptor family, as well as IL10RB, an accessory chain essential for the active interleukin 10 (IL10) receptor complex.

Notably, both CCR1 and CCR5 serve as receptors for the same set of cytokine/chemokine ligands, including macrophage inflammatory protein 1 alpha (MIP-1 alpha), monocyte chemoattractant protein 3 (MCP-3), myeloid progenitor inhibitory factor-1 (MPIF-1), and regulated on activation normal T expressed and secreted protein (RANTES). The latter is well-known as a biomarker of vascular dysfunction in the pulmonary interface and a major driver of hypertension (Nosalski and Guzik, 2017; Funk-Hilsdorf et al., 2022). Its receptor CCR5 is expressed at the surface of T cells and macrophages and serves as a co-receptor for the cell entrance for macrophage-tropic viruses. One single-cell RNA sequencing study of immune-epithelial interactions within the lung tissue (Chua et al., 2020) indicated that during the infection of SARS-CoV-2, the activated resident macrophages in general and inducible ligands for CCR1 and CCR5 in particular, contribute to inflammatory tissue damage, lung injury, and respiratory failure. In addition to CCR1 and CCR5, our study highlighted involvement of IL10RB, whose co-expression with IL10RA is required for IL10-induced signal transduction. IL10 has pleiotropic effects on immunoregulation and inflammation of mucosal tissues. Notably, mucosal integrity and immunity are indispensable for the prevention of symptomatic COVID-19 (Arrieta et al., 2009; Cortes et al., 2022). In a recent genome-wide study, IL10RB was identified as the top key regulator of COVID-19 host susceptibility, with higher IL10RB expression in patient blood being associated with worse COVID-19 outcomes (Voloudakis et al., 2021). Furthermore, in multiple rodent models, the recombinant IL10 can exert direct antihypertensive action by increasing the production of nitric oxide (NO), a kind of potent vasodilator (Lima et al., 2016; Gillis et al., 2020). Incidentally, the same sets of molecular networks have been reported to be involved in the pathogenesis of multiple sclerosis (Szczucinski and Losy, 2007; Porro et al., 2020), one of the diseases highlighted by our ingenuity-driven analysis of canonical pathways.

The strengths of this study include its multi-pronged design, which included two different definitions of severe COVID-19 outcomes as well as three types of hypertension-related parameters. We should also stress that all or a majority of study participants were of European ancestry, thus reducing the potential population heterogeneity. Several limitations should be acknowledged, one is the heterogeneity of our findings. Observed levels of heterogeneity indicate inherent complexity of relationships between hypertension and severe COVID-19. In part, heterogeneity may be explained by differing age and gender structure of studied populations. In addition, severity of COVID-19 may be affected by a large number of other confounders such as BMI, smoking, history of medications, and presence of comorbidities (Cai and He, 2019; Cai and He, 2021). We have also detected a moderate publication bias, possibly explained by a lack of interest in publishing largely negative association trends. A further meta-analysis of larger sets of independent publications is warranted.

In conclusion, our results suggest that elevated blood pressure is significantly associated with the COVID-19 severity. The genetic liabilities to elevated blood pressure and to severe COVID-19 intertwine, and among them, the immune-regulating receptors CCR1/CCR5 and IL10RB signaling pathway are highlighted in the common effective tissue of the lung.

## Data Availability

The original contributions presented in the study are included in the article/Supplementary Material; further inquiries can be directed to the corresponding authors.
